# Causal association between blood and urine biomarkers, immune cells, and bladder cancer: A Mendelian randomization and mediation analysis

**DOI:** 10.1097/MD.0000000000042814

**Published:** 2025-06-06

**Authors:** Feng Lin, Kewei Yang, Tianbo Luo, Tianqi Chen

**Affiliations:** a Department of Urology, Affiliated Hospital of Shaoxing University, Shaoxing, Zhejiang, China.

**Keywords:** bladder cancer, blood and urine biomarkers, immune cells, mediation analysis, Mendelian randomization

## Abstract

Bladder cancer (BC) is influenced by genetic and environmental factors, with blood and urine biomarkers playing a critical role in its diagnosis and progression. However, establishing the causal association between these biomarkers and BC remains challenging due to confounding factors and reverse causation in traditional studies. Therefore, we conducted a Mendelian randomization (MR) analysis to assess the causal association between these biomarkers and BC. A bidirectional MR analysis was performed using pooled data from 35 blood and urine biomarkers, 731 immune cell types, and BC cases from the Genome-Wide Association of Transgenics and Circulating Metabolites study. Complementary analyses, including mediation analysis, 2-stage MR, and multivariate MR, were employed to investigate the potential mediating role of immune cells in this association. We further conducted sensitivity analyses to validate the stability and feasibility of our dataset. The analysis identified a causal association between BC and 2 biomarkers: calcium and sex hormone-binding globulin. Elevated calcium levels were associated with an increased risk of BC (inverse variance weighting: [OR] = 1.295, 95% [CI] = 1.062–1.578, *P *= .011), while higher sex hormone-binding globulin levels were linked to a decreased risk (inverse variance weighting: OR = 0.857, 95% CI = 0.741–0.991, *P *= .037). Notably, CD20 expression on IgD⁻ CD24⁻ B cells appeared to attenuate the positive association between calcium and BC. This study reinforces the association between specific blood and urine biomarkers and the risk of developing BC. It also highlights the mediating role of CD20 on IgD⁻ CD24⁻ B cells in the causal pathways linking these biomarkers to BC. These insights enhance our understanding of BC pathogenesis and may guide the development of targeted diagnostic and therapeutic strategies.

## 1. Introduction

Bladder cancer (BC) is a major urological cancer, ranking as the tenth most common cancer and the thirteenth leading cause of cancer-related mortality worldwide.^[1]^ The Global Burden of Disease 2019 study reported a 54.4% increase in BC incidence and a 30.4% rise in mortality rates over recent decades. According to GLOBOCAN 2020 database, an estimated 573,278 new BC cases and 212,536 deaths occurred in 2020, accounting for 3.0% of all new cancer cases and 2.1% of all cancer-related deaths.^[2]^ In the United States, the incidence is approximately 20.1 per 100,000 men and 4.9 per 100,000 women.^[3]^ BC incidence is also high in Europe, particularly in southern and western regions, as well as in East Asia, where the disease burden remains substantial.^[4,5]^ The predominant histological type of BC is urothelial carcinoma (UC). The etiology of BC has been linked to several factors, including smoking, occupational exposure to industrial chemicals (especially in the dye and rubber industries), and chronic bladder inflammation.^[6]^ Advances in molecular biology have identified several genetic mutations associated with BC, such as FGFR3, TERT, and p53. A study by Pepe et al revealed overexpression of HBA1, HBA2, and SNCA genes in muscle-invasive BC (MIBC) patients.^[7]^ These genetic alterations are crucial in the oncogenesis and progression of BC, significantly influencing treatment response and prognosis. In recent years, noninvasive diagnostic techniques have gained importance. The Bladder EpiCheck methylation assay has proven valuable in diagnosing BC, reinforcing the molecular validity of the Paris System for Reporting Urinary Cytology (TPS) classification and providing complementary evidence to enhance the accuracy of urine cytology diagnosis.^[8]^ Using urine DNA methylation analysis, a higher EpiScore was significantly associated with subsequent risk of BC recurrence.^[9]^ Beyond its direct impact on mortality, BC also contributes to severe comorbidities, including urinary tract irritation, renal insufficiency, hematuria, and cardiovascular disease.^[10,11]^ The co-occurrence of these conditions suggests shared pathogenic pathways and risk factors. Understanding the epidemiology, pathogenesis and wider health impact of BC is essential to develop targeted therapies and improve patient outcomes.

Several biochemical factors contribute to BC tumorigenesis and progression. Elevated serum copper and zinc levels have been implicated in BC progression, with copper promoting angiogenesis, a key factor in tumor growth.^[12]^ In addition, elevated levels of C-reactive protein (CRP), an inflammatory marker, are associated with bladder tumor progression.^[13]^ Moreover, significant alterations in glycosylation patterns have been observed in BC cells, including increased glycoprotein expression and structural modifications such as abnormal sialylation and fucosylation.^[14]^ These changes are closely linked to tumor cell proliferation, invasion and metastasis. Glycoproteins facilitate tumor spread by allowing tumor cells to adhere to and migrate through the extracellular matrix and vascular endothelium.^[15]^ While carcinoembryonic antigen (CEA) is widely used as a biomarker for colorectal cancer, elevated CEA levels have also been detected in certain BC patients, making it a potential surveillance and prognostic marker.^[16]^

A variety of biochemical factors in the tumor microenvironment, such as oxidative stress and inflammatory mediators, play a critical regulatory role in the development of BC.^[17]^ These factors influence tumor growth, invasion and metastasis through the actions of surrounding immune cells, including regulatory T cells (Treg) and myeloid-derived suppressor cells.^[18]^ While some studies suggest that biochemicals may influence BC development and progression by modulating immune cell function, the specific pathways and mechanisms involved require further scientific validation and in-depth research. To overcome the limitations of traditional observational studies, Mendelian randomization (MR) has emerged as a powerful statistical approach for inferring causal relationships between exposure factors and disease outcomes. MR employs genetic variants as instrumental variables (IVs), allowing for better control of confounders.^[19]^ Since genetic variants are determined at birth, they are inherently unaffected by lifestyle factors, environmental exposures, and disease states, providing a robust framework for assessing causal relationships.^[20]^ In this study, we aim to investigate the causal relationship between blood and urine biomarkers and BC, while also exploring the potential mediating role of immune cells in this association.

## 2. Methods

### 2.1. Study design

We employed a 2-stage MR (TSMR) approach to explore the potential mediating role of immune cells between blood and urine biomarkers and BC. First, we used univariate MR (UVMR) to estimate the effect of blood and urine biomarkers on BC. Next, we performed UVMR to assess the effect of immune cells on BC. Finally, UVMR was used to identify immune cells significantly associated with blood and urine biomarkers, and multivariate MR (MVMR) was applied to determine whether these immune cells exhibited a causal relationship with BC after adjusting for blood and urine biomarkers. All analyses were performed in R Studio using R version 4.3.1, with the TwoSampleMR and MVMR packages for the primary analyses.

### 2.2. Data sources

Pooled GWAS data for 35 blood and urine biomarkers were obtained from a meta-analysis of UK Biobanks cohort.^[21]^ A dataset of 35 biomarkers from 363,228 individuals was selected for the study by Nasa Sinnott-Armstrong et al The study included 318,953 White British, 23,582 nonwhite British, 6019 African, 7338 South Asian, and 1082 East Asian individuals. The researchers systematically analyzed the genetic structure of these individuals and performed fine-tuning of biomarker-related loci, including protein-altering variants, protein truncation variants, noncoding variants, HLA variants, and copy number variations. This comprehensive analysis established phenome-wide associations for implicated genetic variants and assessed causal relationships between biomarkers and 40 medically relevant phenotypes. Summary statistics for immune cell phenotypes were obtained from the GWAS database,^[22]^ covering GWAS identifiers GCST90001391 to GCST90002121 (https://www.ebi.ac.uk/gwas/studies/GCST90002121). A cohort study of 3757 Sardinians reported data on 22 million variants across 731 immune cell phenotypes, including 118 absolute cell counts (ACs), 192 relative counts, 389 mean fluorescence intensities of surface antigens, and 32 morphological parameters. Summary statistics for BC in the GWAS dataset were obtained from FinnGen R11, which included 2574 cases and 345,118 controls.^[23]^ FinnGen R11 utilizes advanced genotyping techniques to analyze 220,000 single nucleotide polymorphisms (SNPs) across the genome, leveraging Finland unique population genetics for precise analysis of genetic variations influencing disease.

### 2.3. Selection of instrumental variables

In this study, we used 2-sample and multivariate MR analyses as methods of causal inference to establish the validity of causal effects. The validity of the MR analyses depended on 3 basic assumptions: the IVs are not associated with any confounding variables; the IVs are strongly correlated with the exposure; and the IVs affect the outcomes solely through the exposure.^[24]^ Multivariate analysis was then used to increase the stability of the results. To ensure a sufficient number of SNPs for MR analysis, we followed previously established MR studies. The genome-wide significance threshold for exposure-associated SNPs was set at 5 × 10^−8^. For immune cell and BC-related SNPs, a GWAS threshold of 1 × 10^−5^ was applied. In addition, we used a linkage disequilibrium clustering method (r^2^ = 0.001, window size = 10,000 kb) to remove unwanted SNPs and ensure their independence.^[25]^ We selected SNPs with effect allele frequencies >0.01 and excluded those with *F*-statistics <10 to maintain data quality.^[26]^

### 2.4. Statistical analyses

Mendelian randomization methods were used to assess the causal relationship between blood and urine biomarkers, immune cells and BC. We performed a 2-step MR-mediated analysis to examine whether immune cells act as mediators in the pathway from blood and urine biomarkers to BC. We used various analyses including inverse variance weighted (IVW) analysis, MR-Egger regression, weighted median, weighted mode, and simple mode test. IVW had the highest statistical validity as the primary analytical method, with other methods as further supplements.^[27]^ Results were considered statistically significant if the IVW *P*-value was < .05, and the directionality of IVW and MR-Egger estimates was consistent. In cases where valid IVs were unavailable, the weighted median method was applied, as it provides reliable causal effect estimates even when <50% of the information is derived from valid instruments.^[28]^ If most IVs with similar causal estimates are valid tools, the weighted model approach remains valid even if other IVs do not meet the requirements of MR analysis.^[29]^ In addition to explore whether BC had a causal effect on the identified blood and urine biomarkers, a reverse MR analysis was performed.

### 2.5. Mediation analysis

The mediation proportion was determined based on the formula:(beta1 × beta2)/ beta_all. beta_all represents the total causal effect of blood and urine biomarkers on BC derived from the main analysis, beta1 represents the estimated effect of blood and urine biomarkers on potential immune cells mediators, and beta2 represents the causal effect of immune cells mediators on BC.

### 2.6. Sensitivity analysis

To assess potential horizontal pleiotropy, we examined intercept values from MR-Egger regression, considering nonzero intercepts with *P*-values <.05 as significant indicators of genetic pleiotropy. MR-PRESSO was used to detect horizontal pleiotropy and outliers, and results affected by these were not used in subsequent analyses.^[30]^ We assessed data heterogeneity using Cochran *Q* test and obtained more conservative and robust estimates by applying random-effects IVW analysis.^[31]^ In addition, we performed leave-one-out sensitivity analyses to determine whether individual SNPs significantly influenced the overall causal association.^[32]^

## 3. Results

**Figure 1.**
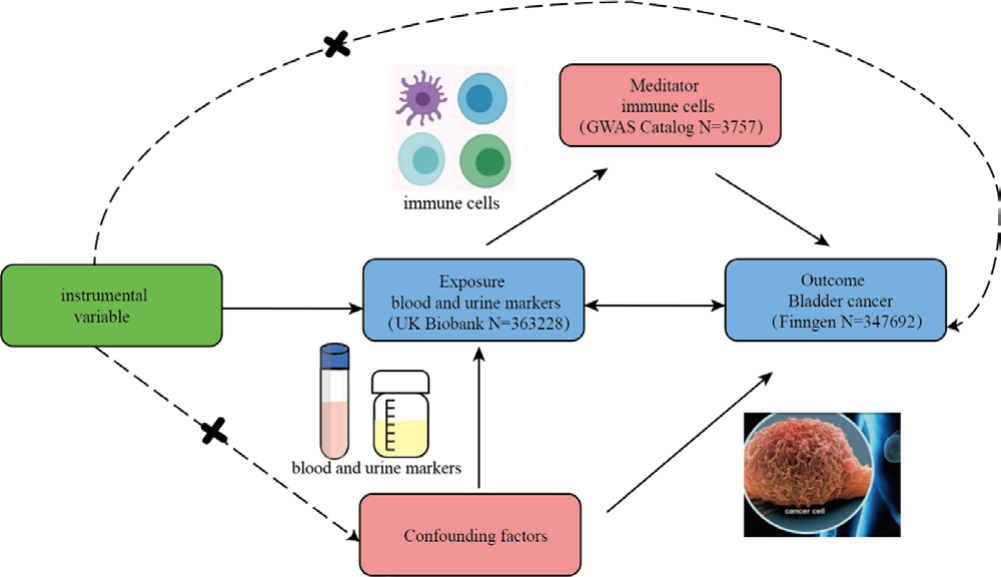
Hypothesis and design of bidirectional and mediated MR analyses. MR = Mendelian randomization.

Figure 1 depicts the flowchart of the study.

### 3.1. Two-sample bidirectional MR analysis of blood and urine biomarkers and BC

**Figure 2.**
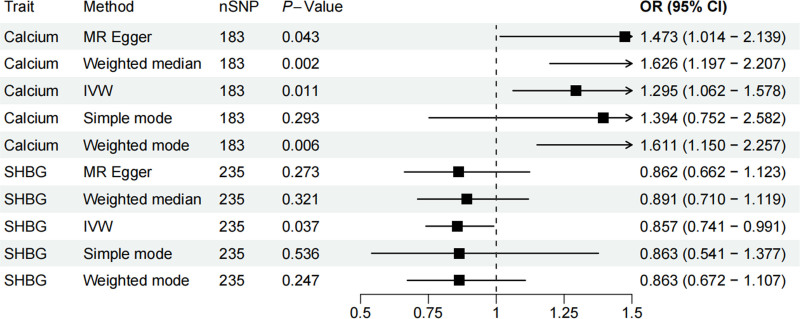
Forest plots depicting the causal impacts of blood and urine biomarkers on BC. BC = Bladder cancer.

**Figure 3.**
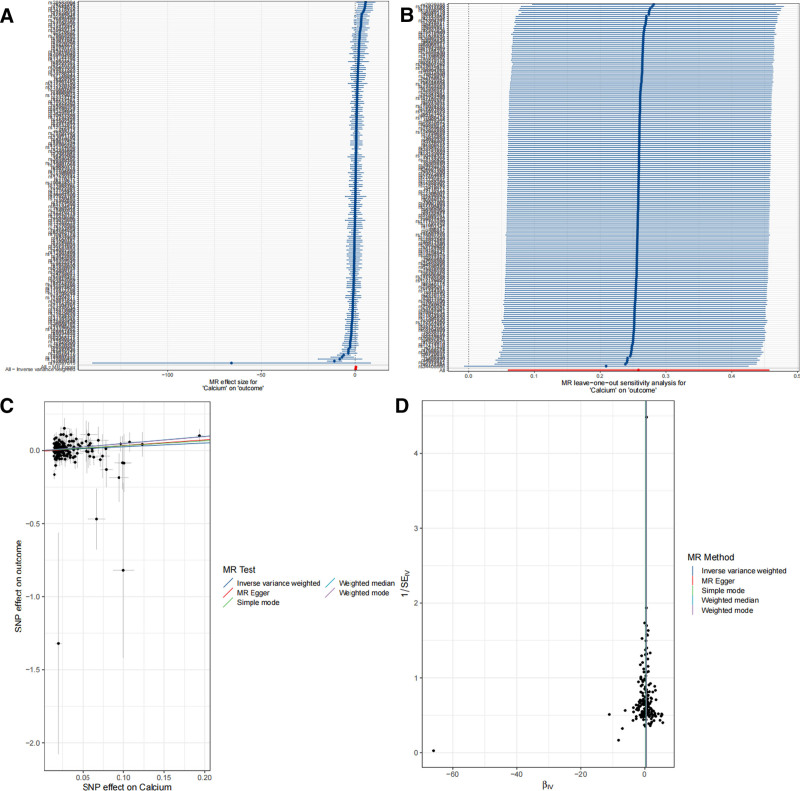
Sensitivity analysis of the causal relationship between calcium and BC: (A) forest plots; (B) leave-one-out plots; (C) scatter plots; (D) funnel plots. BC = bladder cancer.

**Figure 4.**
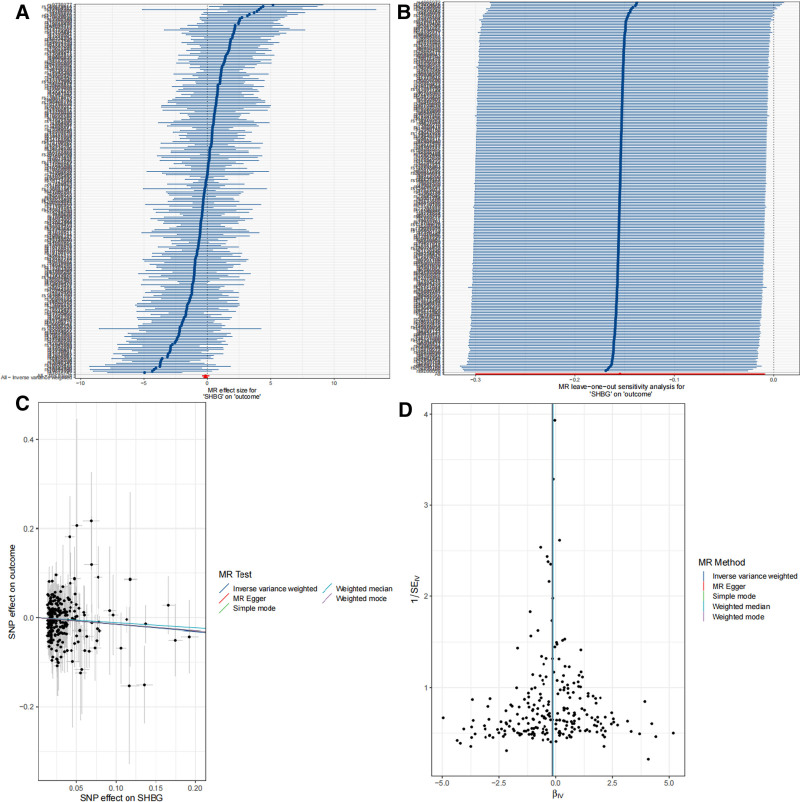
Sensitivity analysis of the causal relationship between SHBG and BC: (A) forest plots; (B) leave-one-out plots; (C) scatter plots; (D) funnel plots. BC = bladder cancer, SHBG = sex hormone-binding globulin.

Mendelian randomization analysis revealed a causal relationship between 2 blood and urine biomarkers and BC, including calcium and sex hormone-binding globulin (SHBG) (Fig. 2). Specifically, we observed that calcium (OR = 1.295, 95% CI = 1.062–1.578; *P *= .011) was associated with an increased risk of BC, while SHBG (OR = 0.857, 95% CI = 0.741–0.991; *P *= .037) was associated with a decreased risk of developing BC. In sensitivity analyses (Figs. 3 and 4), the MR-Egger intercept test did not detect a significant horizontal pleiotropic effect. Even when the results suggested the presence of heterogeneity (*P *= .0027), we preferred to use random-effects IVW analyses to maintain the robustness of the association between blood and urine biomarkers and risk of BC. Leave-one-out analysis did not reveal any confounding by individual SNPs. To investigate whether BC had a causal effect on the identified blood and urine biomarkers, we then performed a reverse analysis. We considered BC-associated SNPs as IVs, BC as exposure, and blood and urine biomarkers as outcome, and we found no reverse causality. In addition, the *F*-statistics of these SNPs are >10, indicating that our results are unlikely to be biased by weak instrumentation.

### 3.2. The overall causal effect of immune cells on BC

**Figure 5.**
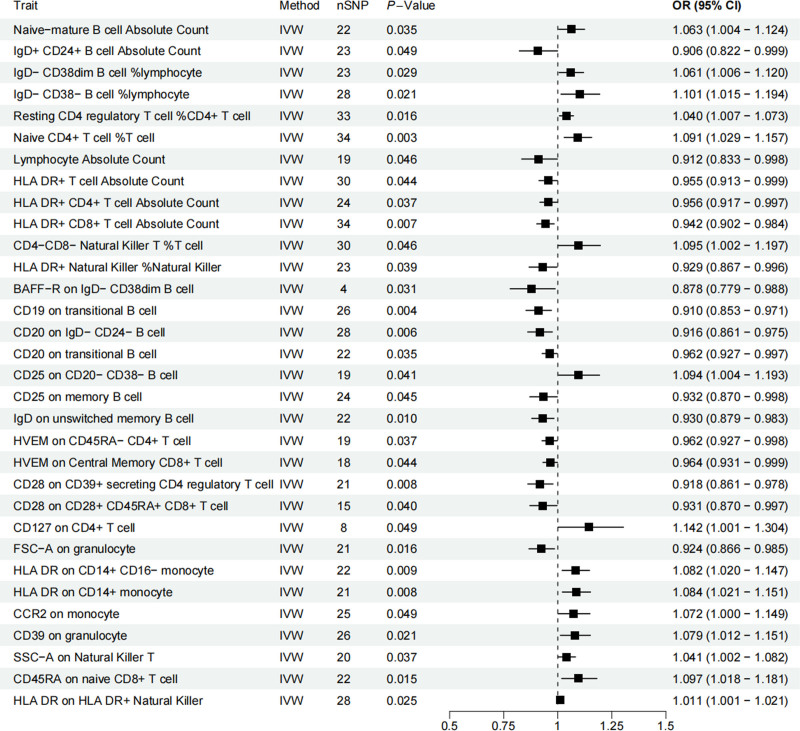
Forest plots depicting the causal impacts of immune cells on BC. BC = bladder cancer.

The IVW results showed a causal relationship between 32 immune cells and BC (Fig. 5), with MR-Egger test showing no significant horizontal pleiotropy. Among these, 27 were lymphocyte subtypes, including 11 B-cell subtypes, 11 T-cell subtypes, 2 NK-cell subtypes, and 2 NKT-cell subtypes. Additionally, 2 granulocyte and 3 monocyte subtypes were also implicated. Notably, several immune cell subtypes were found to increase BC risk, including naive CD4^+^ T cells (OR = 1.091, 95% CI = 1.029–1.156; *P* = .003) and HLA-DR on CD14^+^ monocytes (OR = 1.084, 95% CI = 1.020–1.151; *P* = .008). Conversely, certain B-cell subtypes exhibited protective effects, such as CD20 on IgD⁻ CD24⁻ B cells (OR = 0.916, 95% CI = 0.860–0.975; *P* = .005) and CD19 on transitional B cells (OR = 0.909, 95% CI = 0.852–0.970; *P* = .004), both of which were associated with a reduced BC risk. Sensitivity analyses confirmed that no significant heterogeneity or horizontal pleiotropy was present in these associations (Table 1).

**Table 1:** Heterogeneity test and horizontal pleiotropy test of the association between immune cell and BC.

Exposure	Outcome	Heterogeneity test				Pleiotropy test	
		MR-Egger		IVW		MR-Egger	
		*Q*	*P*	*Q*	*P*	Egger_intercept	*P*
Naive-mature B cell absolute count	BC	21.906	.345	23.836	.301	21.906	.345
IgD + CD24+ B cell absolute count	BC	21.301	.440	21.948	.462	−0.013	.433
IgD^−^ CD38dim B cell % lymphocyte	BC	20.775	.472	21.444	.493	−0.009	.422
IgD^−^ CD38^−^ B cell % lymphocyte	BC	24.818	.529	26.002	.518	−0.144	.285
Resting CD4 regulatory T cell % CD4^+^ T cell	BC	30.843	.474	31.318	.500	0.008	.495
Naive CD4^+^ T cell %T cell	BC	26.038	.761	27.939	.717	0.015	.177
Lymphocyte Absolute Count	BC	19.649	.292	19.939	.336	0.008	.622
HLA-DR + T cell Absolute Count	BC	20.911	.829	22.444	.801	0.014	.225
HLA-DR + CD4^+^ T cell Absolute Count	BC	15.064	.859	15.440	.878	−0.006	.545
HLA-DR^+^ CD8^+^ T cell absolute count	BC	23.449	.863	24.503	.857	0.010	.312
CD4^−^ CD8^−^ Natural Killer T %T cell	BC	36.904	.120	37.055	.144	0.007	.737
HLA-DR^+^ Natural Killer %Natural Killer	BC	19.326	.564	20.013	.582	−0.012	.416
BAFF-R on IgD^−^ CD38dim B cell	BC	0.066	.967	0.169	.982	−0.007	.778
CD19 on transitional B cell	BC	27.050	.302	29.383	.248	0.019	.163
CD20 on IgD^−^ CD24^−^ B cell	BC	21.890	.694	21.891	.742	<0.001	.976
CD20 on transitional B cell	BC	29.832	.072	32.257	.055	0.016	.216
CD25 on CD20^−^ CD38^−^ B cell	BC	20.578	.245	21.924	.235	−0.018	.306
CD25 on memory B cell	BC	32.700	.066	32.728	.085	−0.002	.892
IgD on unswitched memory B cell	BC	19.429	.494	20.018	.520	0.010	.451
HVEM on CD45RA^−^ CD4^+^ T cell	BC	19.303	.311	19.657	.352	0.007	.584
HVEM on Central Memory CD8^+^ T cell	BC	13.187	.659	13.369	.711	−0.006	.675
CD28 on CD39^+^ secreting CD4 regulatory T cell	BC	23.718	.207	24.030	.241	−0.007	.622
CD28 on CD28^+^ CD45RA^+^ CD8^+^ T cell	BC	13.678	.396	14.693	.399	−0.018	.343
CD127 on CD4^+^ T cell	BC	4.021	.673	4.089	.769	−0.009	.802
FSC-A on granulocyte	BC	14.248	.769	15.316	.758	0.016	.314
HLA-DR on CD14^+^ CD16^−^ monocyte	BC	18.603	.547	19.789	.534	0.021	.289
HLA-DR on CD14^+^ monocyte	BC	19.975	.275	21.551	.252	−0.018	.262
CCR2 on monocyte	BC	13.628	.914	14.461	.912	0.012	.371
CD39 on granulocyte	BC	16.404	.872	16.546	.897	−0.005	.709
SSC-A on natural killer T	BC	13.869	.737	13.929	.787	0.002	.809
CD45RA on naive CD8^+^ T cell	BC	15.493	.747	18.529	.615	−0.031	.096
HLA-DR on HLA-DR + natural killer	BC	26.783	.420	26.956	.466	−0.003	.685

BC = bladder cancer, CI = confidence intervals, IVs = instrumental variables, IVW = inverse variance weighting, MR = Mendelian randomization.

### 3.3. Mediation analysis of potential immune cells

**Figure 6.**
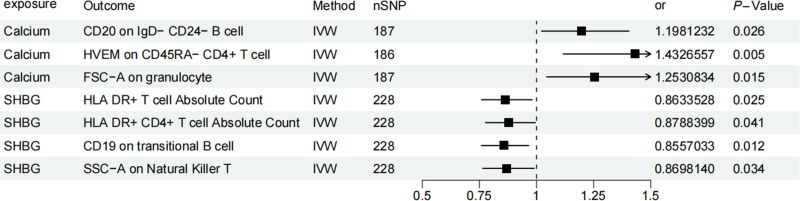
Forest plots depicting the causal impacts of blood and urine biomarkers on immune cells.

**Figure 7.**
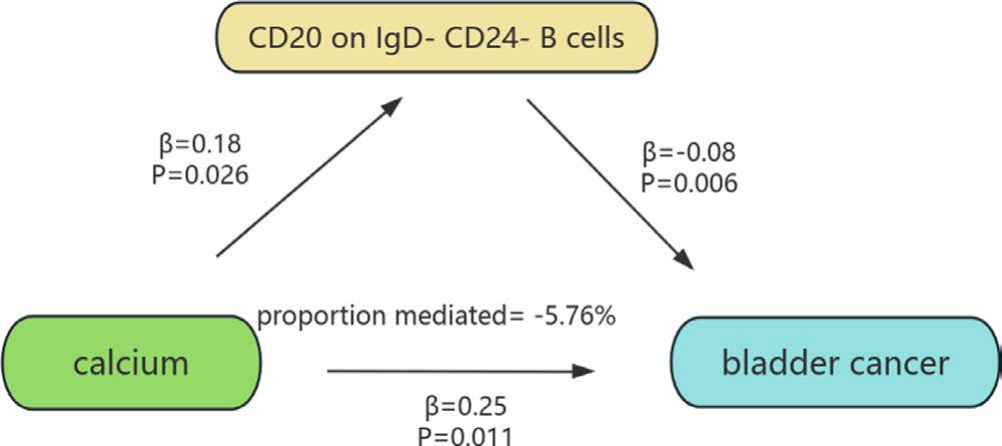
The figure shows the mediation pattern of “calcium-CD20 on IgD^−^ CD24^−^ B cells-BC” in a TSMR. β indicates the estimate of the causal efect using an IVW method (*P* < .05). BC = bladder cancer.

Our MR studies described above have identified blood and urine biomarkers and immune cells associated with BC. We performed a mediation analysis in order to determine the mediating role of immune cells between blood and urine biomarkers to BC. First, we assessed the relationship between blood and urine biomarkers and immune cells using 2-sample MR, identifying associations between 7 blood and urine biomarkers and immune cells (Fig. 6): association of calcium and CD20 on IgD^−^ CD24^−^ B cell (OR = 1.198, 95% CI = 1.021–1.405; *P* = .026), association of calcium and HVEM on CD45RA^−^ CD4^+^ T cell (OR = 1. 432, 95% CI = 1.116–1.838; *P* = .004), association of calcium and FSC-A on granulocyte (OR = 1.253, 95% CI = 1.044–1.503; *P* = .015), association of SHBG and FHLA DR^+^ T-cell absolute count (OR = 0. 863, 95% CI = 0.759–0.981; *P* = .024), association of SHBG and HLA-DR^+^ CD4^+^ T-cell absolute count (OR = 0.878, 95% CI = 0.776–0.994; *P* = .041), association of SHBG and CD19 on transitional B cells (OR = 0. 855, 95% CI = 0.758–0.965; *P* = .011), association of SHBG and SSC-A on Natural Killer T (OR = 0.869, 95% CI = 0.764–0.989; *P* = .034). These results showed no evidence of heterogeneity and horizontal pleiotropy. Finally, we also used MVMR to assess the independent effects of the 7 mediators on BC. It was found that only CD20 on IgD^−^ CD24^−^ B cells showed a significant negative mediating effect between calcium and BC after adjustment for blood and urine biomarkers. The percentages indicating CD20 on IgD^−^ CD24^−^ B-cells mediation were found to be −5.76% (Fig. 7).

## 4. Discussion

Using MR analysis, this study provides stronger evidence of a causal relationship between blood and urine biomarkers and BC. This type of analysis helps to reduce the problems of confounding and reverse causation that are common in traditional observational studies. In this study, we found that calcium was strongly associated with an increased risk of BC (OR = 1.295, 95% CI = 1.062–1.578, *P* = 0. 011), whereas SHBG was associated with a decreased risk of BC, which is a protective factor against the development of BC (OR = 0.857, 95% CI = 0.741–0.991; *P* = .037).

Sex hormone-binding globulin is a plasma glycoprotein that acts on hormone-responsive tissues by regulating the action and bioavailability of steroid hormones.^[33]^ Several epidemiologic studies have suggested that SHBG levels may be associated with the risk of certain cancers, such as prostate cancer.^[34]^ Several studies have shown that higher SHBG levels are negatively correlated with breast cancer risk.^[35]^ Mechanistically, SHBG inhibits estradiol-mediated ERK activation, leading to reduced cell growth and antiapoptotic effects.^[36]^ It has been shown that androgen receptor (AR) has a high positivity rate in bladder UC, suggesting that UC may also be a hormone-dependent carcinoma.^[37]^ The expression of AR decreases with the histologic grading of UC, and it is possible that AR is a negative regulator of the proliferation of bladder UC.

Several studies have shown that high calcium intake may be associated with a lower risk of certain cancers, but this association is inconclusive for BC.^[38]^ Cellular Ca^2+^ homeostasis is regulated by multiple ion channels, including transient receptor potential channels and store-operated channels, which mediate extracellular Ca^2+^ influx. Dysregulation of these calcium channels, as well as mitochondrial calcium buffering, disrupts intracellular signaling and contributes to oncogenic transformation.^[39]^ Transient receptor potential channels are classified into different subfamilies and are associated with a variety of cancers, and downregulation of TRPV4 expression levels facilitates BC.^[40]^ Activation of TRPV1 by capsaicin and activation of TRPV2 by cannabidiol produces a sustained influx of intracellular Ca^2+^, which induces apoptosis in prostate and BC cells, respectively.^[41]^ Voltage-gated calcium channels are the main players in the Ca^2+^ entry mechanism in excitable cells. One of these subunits, CaV1.3, is highly expressed in BC.^[42]^ Therefore, researchers are exploring the therapeutic potential of targeting calcium channels as a strategy to restore calcium homeostasis, enhance cancer cell susceptibility to apoptosis, and ultimately improve treatment efficacy. Epidemiological studies have reported that elevated total and ionized calcium levels are significantly correlated with prostate cancer risk, particularly for ionized calcium, although further validation is needed.^[43]^ In a study that focused on analyzing the effects of various micronutrients such as calcium and phosphorus on BC risk, and a positive association between calcium intake and BC was observed. Participants with lower magnesium intake and higher calcium and phosphorus intake had an increased risk of developing BC.^[44]^ Our MR findings further confirm a strong positive association between elevated calcium levels and BC risk, suggesting that calcium signaling dysfunction may promote tumor initiation and progression by modulating cell signaling, adhesion, and intercellular communication.^[45]^ High calcium levels promote uncontrolled cell division by activating cell signaling pathways, which is a major driver of tumor growth. Calcium ions regulate the cell cycle. When calcium signaling is dysregulated, cancer cells avoid apoptosis, thereby gaining a survival advantage that promotes cancer cell expansion and the accumulation of malignant mutations. Additionally, calcium plays a dual role in autophagy regulation, with elevated calcium levels enhancing autophagy, thereby allowing cancer cells to survive under unfavorable conditions.^[46]^

In the tumor microenvironment (TME), tumor-infiltrating B cells (TIL-B) are mostly found in the vicinity of various T-cell populations, NK cells and myeloid cells, which can lead to the formation of ectopic immune cell aggregates. These form tertiary lymphoid structures and play a central role in the initiation and maintenance of adaptive immune responses.^[47]^ Monocytes, such as macrophages and dendritic cells, are normally present in the human bladder and can be additionally recruited from the circulation. Macrophages in particular are usually polarized to a pro-tumorigenic “M2” phenotype in BC, which is associated with tumor angiogenesis and tissue remodeling and may promote tumor growth and metastasis.^[48]^ Our results confirm that 32 immune cell subtypes exhibit a causal relationship with BC, with lymphocytes constituting the majority, particularly B cells, T cells, and NK cells, followed by granulocytes and monocytes. B cells can provide stronger signals to T cells, such as the inducible co-stimulatory factor (ICOS) ligands CD80 and CD86, to support T-cell function in immune killing. The expression of B7-H4 and PD-L1 in CD8^+^ T cells and CD4^+^ T cells was significantly increased in the circulation and tumor tissue of BC patients. When both B7-H4 and PD-L1 were blocked, the functions of both T cells were restored, as evidenced by an increase in interferon gamma (IFN-γ) and granzyme B production. Thus, B7-H4 not only plays an important role in BC progression, but may also be a new target for future BC treatment.^[49]^ High levels of tumor-infiltrating T cells (TILs) are correlated with better treatment response and improved prognosis in BC.^[50]^ While B cells are generally associated with antitumor immunity, certain subsets, such as tumor-associated regulatory B cells (Bregs), infiltrate BC tissues at significantly higher levels than in adjacent noncancerous tissues and correlate with increased tumor malignancy. Bregs, through the secretion of immunosuppressive cytokines (e.g., IL-10, TGF-β, etc) help to create a microenvironment that suppresses the immune response. These cytokines inhibit the activation and function of effector T cells, thereby helping tumor cells evade immune surveillance.^[51]^ Our study demonstrated that CD20^+^ B cells and CD8^+^ T cells play protective roles in BC, including CD20 on IgD^−^ CD24^−^ B cells, CD20 on transitional B cells, and HLA-DR^+^ CD8^+^ T cells, whereas other B-cell subsets, such as naive-mature B cells and IgD^−^ CD38dim B cells, were associated with increased BC risk. Our study also confirmed that monocytes also tend to promote the generation of BC, such as HLA-DR on CD14^+^ CD16^−^ monocyte, HLA-DR on CD14^+^ monocyte, and CCR2 on monocyte.

In this study, a negative mediated effect was found between calcium and BC through the immune cell CD20 on IgD^−^ CD24^−^ B cells. CD20 is a transmembrane protein expressed by B lymphocytes, and increased Ca^2+^ concentrations have been shown to stimulate CD20 gene transcription and translation, leading to increased surface expression. Elevated calcium levels can activate a number of transcription factors, the most important of which is NFAT (nuclear factor-activated T cells).^[52]^ Calcium ions bind to calmodulin, forming a complex that subsequently activates NFAT, causing it to translocate from the cytoplasm to the nucleus. Once in the nucleus, NFAT binds to a specific sequence in the promoter region of the CD20 gene, initiating transcription of the CD20 gene.^[53]^ A study by Tedder et al suggested that CD20 plays a key role in the activation and proliferation of B cells. CD20 promotes B-cell proliferation and activation by driving B cells from G1 to S phase.^[54]^ Calcium signaling in response to CD20 also promotes the process of differentiation of B cells into plasma cells (PCs), which in turn increases antibody production.^[55]^ Our mediation analysis further confirms the beneficial role of CD20^+^ B cells in BC suppression. The presence of CD20 indicates that these cells are likely still at the mature B-cell stage rather than fully differentiated into PCs that do not express CD20.^[56]^ CD20^+^ B cells and CD8^+^ T cells interact via co-stimulatory signaling pathways (e.g., CD40/CD40L).^[57]^ CD20^+^ B-cells recruit CD8^+^ T cells by releasing chemokines, and then the B-cell population attracts T cells in inflammatory responses and immune cell interactions. In turn, helper T cells also induce B cells to differentiate into PCs and produce antibodies in response to antitumor responses.^[58]^ Activated memory B cells can lose IgD expression, and CD24^−^ B cells may represent a mature or activated B-cell subset.^[59]^ Thus, among CD20 on IgD^−^ CD24^−^ B cells, which tend to be predominantly memory or activated mature B cells, they are likely to influence B cell activation and proliferation by modulating transmembrane calcium conductance, thereby reducing the risk of BC.

This study is the first to utilize a comprehensive MR framework to explore causal associations between blood and urine biomarkers, immune cells, and BC, effectively reducing confounding bias and eliminating reverse causality concerns. Nevertheless, this study has some limitations, and MR analysis could not completely exclude all confounding factors. The data used in this study were primarily from specific ethnic groups (e.g., people of European ancestry), and the findings may not be fully applicable to other ethnic groups or populations, which limits the external validity of the results. In addition, the study identified potential causal relationships between blood and urine biomarkers and BC, but the biological mechanisms and pathways of these biomarkers may be complex and incompletely understood, and further biological studies are needed to validate and provide insight into these relationships. Finally, studies have focused on specific biomarkers and immune cells, and future studies will involve a wider range of biomarkers (e.g., urinary exosomes, IL-6, UCA1) and immune cell subsets (e.g., M2-type macrophages, depleted T cells) to fully assess their role in BC pathogenesis.

## 5. Conclusions

In this study, we utilized MR analysis to strengthen the causal relationship between blood and urine biomarkers and BC, particularly highlighting the associations of calcium and SHBG with BC risk. We found that calcium levels were significantly associated with an increased risk of BC, whereas increased SHBG levels were associated with a decreased risk of BC. In addition, through mediation analysis, we found that immune cells, particularly CD20 on IgD^−^ CD24^−^ B cells, play a significant negative mediating role between calcium and BC risk, providing new biological insights into how calcium affects BC through immune pathways.

### Acknowledgments

We sincerely express our gratitude to all investigators and participants of the GWAS and biobank studies whose summary statistics were utilized in this study.

## Author contributions

**Conceptualization:** Feng Lin.

**Data curation:** Tianbo Luo.

**Formal analysis:** Tianbo Luo.

**Investigation:** Feng Lin.

**Methodology:** Feng Lin.

**Resources:** Kewei Yang.

**Software:** Tianqi Chen.

**Supervision:** Kewei Yang.

**Writing – original draft:** Feng Lin.

**Writing – review & editing:** Tianqi Chen.
